# Melatonin and Bacterial Cellulose Regulate the Expression of Inflammatory Cytokines, VEGF, PCNA, and Collagen in Cutaneous Wound Healing in Diabetic Rats

**DOI:** 10.3390/polym16182611

**Published:** 2024-09-15

**Authors:** Jaiurte Gomes Martins da Silva, Ismaela Maria Ferreira de Melo, Érique Ricardo Alves, Glícia Maria de Oliveira, Anderson Arnaldo da Silva, Isabela Macário Ferro Cavalcanti, Diego Neves Araujo, Flávia Cristina Morone Pinto, José Lamartine de Andrade Aguiar, Valéria Wanderley Teixeira, Álvaro Aguiar Coelho Teixeira

**Affiliations:** 1 Graduate Program of Animal Bioscience, Department of Animal Morphology and Physiology, Federal Rural University of Pernambuco, Recife 52171-900, PE, Brazil; 2 Department of Medicine, Federal University of Alagoas, Arapiraca 57309-005, AL, Brazil; 3 Graduate Program of Therapeutic Innovation, Department of Biochemistry, Federal University of Pernambuco, Recife 50170-901, PE, Brazil; 4 Graduate Program in Biosciences and Biotechnology in Health, Oswaldo Cruz Foundation, Federal University of Pernambuco, Recife 50170-901, PE, Brazil; 5 Laboratory of Clinical Microbiology, Keizo Asami Institute (iLIKA), Federal University of Pernambuco (UFPE), Recife 50170-901, PE, Brazil; 6 Graduate Program of Surgery, Department of Surgery, Federal University of Pernambuco, Recife 50170-901, PE, Brazil

**Keywords:** pineal, streptozotocin, inflammation, dressings

## Abstract

The poor healing of diabetic wounds is characterized by prolonged inflammation and decreased collagen deposition. Diabetic patients exhibit changes in the plasma concentrations of pro-inflammatory cytokines, and the role of specific dressings may have an impact on healing. This study aims to evaluate the effects of a combined treatment comprising a bacterial cellulose dressing and melatonin application on the regulation and expression of inflammatory cytokines, VEGF, PCNA, and collagen in the healing of cutaneous wounds of diabetic rats. Pro-inflammatory cytokines, including IL-6, TNF-α, and VEGF, along with PCNA and type I and III collagen, were evaluated after 14 days. Immunohistochemistry showed decreased levels of IL-6, TNF-α, and VEGF, along with an increased expression of PCNA and type I collagen, in the groups treated exclusively with melatonin and bacterial cellulose associated with melatonin compared to the control and the commercial healing agent. It was concluded that treating the skin lesions of diabetic animals supplemented with melatonin using a bacterial cellulose-based dressing has positive effects in regulating the expression of inflammatory cytokines, vascular endothelial growth factor, and collagen, showing that this association could be a viable therapy approach in wound healing.

## 1. Introduction

Wound healing is a complex and dynamic physiological process triggered by a cellular response to injury and composed of overlapping and orchestrated phases, such as hemostasis, inflammation, proliferation, and remodeling. Each of these phases is tightly regulated by the interplay between cells such as keratinocytes, fibroblasts, leukocytes, macrophages, endothelial cells, and platelets. These cells release an array of growth factors and cytokines, such as transforming growth factor-beta (TGF-β), vascular endothelial growth factor (VEGF), interleukin-6 (IL-6), and tumor necrosis factor-alpha (TNF-α), which coordinate the progression of the wound healing process and ensure successful tissue repair [1,2,3].

Diabetes mellitus (DM) is a chronic metabolic disorder characterized by persistently high blood glucose levels, which lead to various systemic and local complications. Diabetic microangiopathy is one of the most common complications of hyperglycemia. This vascular damage compromises the skin’s barrier function and delays wound healing. The poor healing of diabetic wounds is mainly characterized by prolonged inflammation, which disrupts normal progression through the healing phases and leads to stagnation of the proliferative phase. Furthermore, the extracellular matrix in diabetic wounds tends to exhibit reduced collagen deposition, a factor critical for wound strength and tissue remodeling [4,5,6]. These changes in the healing process of diabetic patients are compounded by imbalances in the plasmatic concentrations of pro-inflammatory cytokines such as IL-6, TNF-α, and VEGF, which further exacerbate the impaired healing response in diabetic individuals [7,8].

Diabetic wounds bring great pain to patients and impose an economic burden on individuals and society, thus requiring treatment strategies that are more effective and less expensive [9,10]. Bacterial cellulose (BC)-based dressings are an affordable and effective strategy for the treatment of skin lesions [11,12,13,14], as are melatonin-based products that have proven effects on healing [15,16,17,18,19].

Therefore, this study aims to evaluate the effects of an innovative, accessible, and effective dressing produced based on bacterial cellulose and used in conjunction with melatonin and to describe its effects on the regulation and expression of inflammatory cytokines, VEGF, PCNA, and collagen in the healing of cutaneous wounds in an experimental model of rats with diabetes.

## 2. Method

### 2.1. Experimental Design

Albino rats (*Rattus norvegicus albinus*) of the Wistar lineage, 60 days old, weighing approximately 250 ± 30 g, from the vivarium of the Department of Animal Morphology and Physiology (DMFA) of the Federal Rural University of Pernambuco (UFRPE) were used. The animals were maintained in an environment with a controlled temperature (22 ± 1 °C) and photoperiod (12 h light and 12 h dark), and food and water were provided ad libitum.

The animals were evaluated daily, during an experimental period of 14 days and randomly divided into 4 groups, with 4 animals in each, as described below:

GC—control group with non-diabetic rats; GDCC—diabetic rats treated with a commercial healing agent; GDCB—diabetic rats treated with bacterial cellulose; and GDMCB—diabetic rats treated with melatonin and bacterial cellulose.

This study followed the principles established by the Brazilian laws on the use and creation of animals (n° 11.794/2008), which regulate research with animals in Brazil. The study was approved by the Ethics Committee of the Use of Animals of the Federal Rural University of Pernambuco (CEUA—UFRPE) under registration no. 052/2019.

### 2.2. Diabetes Induction

Induction was performed by intraperitoneal administration of a streptozotocin solution (Sigma Chemical Co., St. Louis, MO, USA) after a 14 h fasting period. Streptozotocin was diluted in 10 mM sodium citrate buffer with pH 4.5, and a single dose consisted of 60 mg/kg of animal weight. The non-diabetic animals received equivalent doses of saline solution. After 30 min, all animals were fed normally. Diabetes was confirmed 5 days after induction. Only animals that presented glycemia above 200 mg/dL (Accu-Chek Activ Kit glucometer, Accu-Chek, Singapore) were included in the study, except for the control group [20].

### 2.3. Melatonin Treatment

Melatonin, N-acetyl-5-methoxytryptamine (Sigma Chemical Co., St. Louis, MO, USA), was administered in daily injections intraperitoneally from 6:00 p.m. to 7:00 p.m. at 10 mg/kg; it was dissolved in 0.2 mL of ethanol and diluted in 0.9 mL of 0.9% NaCl.

### 2.4. Treatment with Bacterial Cellulose and Commercial Healing

A bacterial cellulose (BC) hydrogel and a commercial healing agent (CC) were applied directly to each animal’s wound once a day with a dose of approximately 0.4 mL. The commercial healing agent used was an amorphous hydrogel with alginate, chosen because of its wide use and similarities to the BC hydrogel regarding physicochemical characteristics. The BC hydrogel was produced from sugarcane molasses at the Sugarcane Experimental Station (EECC) of the Federal Rural University of Pernambuco (UFRPE) and supplied by the company POLISA^®^, specializing in biopolymers for health. Bacterial cellulose is produced by propagating *Gluconacetobacter hansenii* in a sterile culture obtained from sugarcane molasses. The polymeric mass is mechanically processed to obtain the gel and, subsequently, the hydrogel. All stages of production are carried out in accordance with the standard operating procedure, a process developed and patented by Polisa Biopolímeros para Saúde Ltda. Samples of BC hydrogel dressings tested in this study were prepared from a 0.7% concentration.

### 2.5. Immunohistochemistry

For immunohistochemical analysis, silanized slides were deparaffinized and rehydrated in xylene and alcohols. Antigenic retrieval was performed using a citrate buffer solution (pH 8.0) at a high temperature in the microwave for 5 min. Endogenous peroxidase was inhibited using a solution of hydrogen peroxide (3%) in methanol. The nonspecific antigen–antibody reaction was blocked by incubating the slides in PBS and 5% bovine serum albumin (BSA) for one hour. IL-6 (sc-32296, Santa Cruz Biotechnology, Santa Cruz, CA, USA), TNFα (sc-33639, Santa Cruz Biotechnology, Santa Cruz, CA, USA), VEGF (MBS2540134, MyBioSource, San Diego, CA, USA), and PCNA (Santa Cruz Biotechnology, Santa Cruz, CA, USA) antibodies were diluted 1:50 in 1% PBS/BSA and incubated for 1 h. Subsequently, the slides were treated with the secondary antibody for 30 min. The antigen–antibody reaction was observed through a brown precipitate after application of 3,3′-diaminobenzidine for four minutes and counterstained with hematoxylin. Images of the IL6, TNFα, and VEGF slides were captured using a Sony^®^ Video camera, coupled to the Olympus^®^ Bx50 microscope, and the images were submitted to the Gimp 2.0 application for quantification through the use of an RGB Histogram (red–green–blue) [21,22].

For PCNA, cell counts were performed using a Weibel graticule with 25 observation points in a 10× eyepiece. Three slides per group were used, and four fields/slide were analyzed. In each field, 300 cells were counted and transformed into percentage of marked cells [23].

### 2.6. Evaluation and Quantification of Collagen I and III

For the evaluation and quantification of type I and III collagen, the sections were stained using the Picrosirius Red histochemical technique. After mounting with Entellan^®^, the slides were photomicrographed under polarized light in an Axio microscope (Imager.M2m/Zeiss, Oberkochen, Germany) with an AxioCam camera (HRc/Zeiss) attached. Five images per section were obtained under 200× magnification and processed using the ImageJ^®^ software version 1.52, where color markings were made by selecting the corresponding pixels in all images. In this way, the percentage of area occupied in the image by each of the two markings was obtained. For collagen type I, pixels were marked in red, and for type III, pixels were marked in green [24].

## 3. Results

During the experimental period of this study, there were no animal losses, and no complications, including procedure-related infection, were observed.

### 3.1. Immunohistochemistry (IL-6, TNF-α, VEGF, and PCNA)

**Figure 1:**
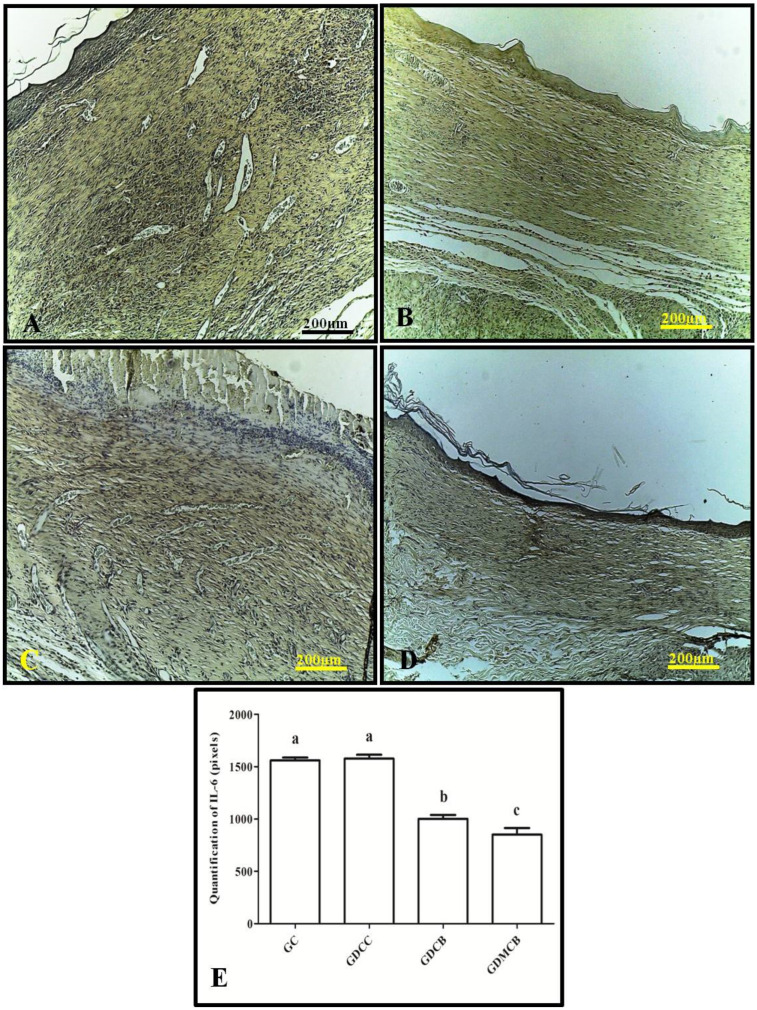
Immunohistochemical analysis for IL-6 of wounds from animals at day 14. (**A**) GC group; (**B**) GCC group; (**C**) GDCB group; (**D**) GDMCB group. There is a higher expression in the GC and GDCC groups and a lower expression in the GDMCB group. (**E**) Quantification in pixels. Means with the same letter do not differ significantly from each other, as determined via Tukey and Kramer’s multiple comparisons test (*p* > 0.05).

Immunohistochemistry for IL-6 in the wounds showed weak staining after 14 days in the GDCB and GDMCB groups, whereas the GC and GDCC groups showed a strong staining pattern. Regarding the quantification of IL-6 expression in pixels, we observed a significant decrease in pixels in the GDMCB group compared to the other groups (Figure 1).

**Figure 2:**
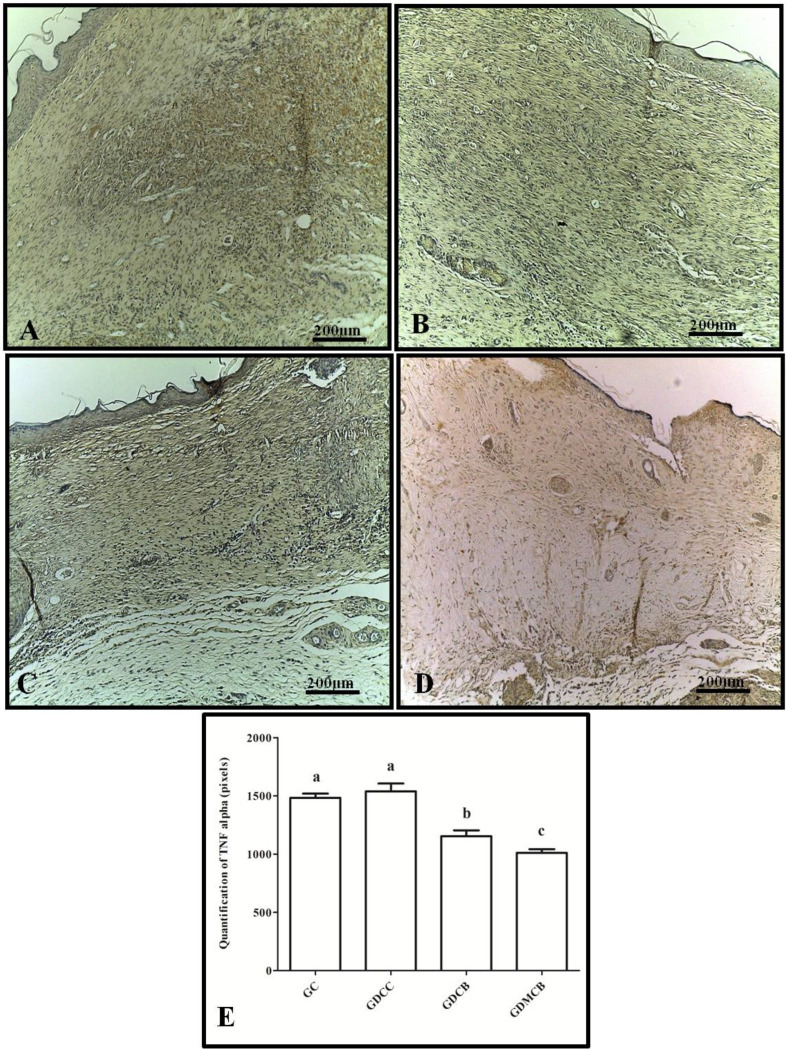
Immunohistochemical analysis for TNFα of wounds from animals at day 14. (**A**) GC group; (**B**) GCC group; (**C**) GDCB group; (**D**) GDMCB group. There is a higher expression in the GC and GDCC groups and a lower expression in the GDMCB group. (**E**) Quantification in pixels. Means with the same letter do not differ significantly from each other, as determined via Tukey and Kramer’s multiple comparisons test (*p* > 0.05).

The analysis of TNF-α in the wounds revealed weak staining after 14 days in the GDCB and GDMCB groups, whereas the GC and GDCC groups showed a strong staining pattern, but no statistical difference was found. Regarding quantification of TNF-α expression in pixels, we observed a significant decrease in pixels in the GDMCB group compared to the other groups (Figure 2).

**Figure 3:**
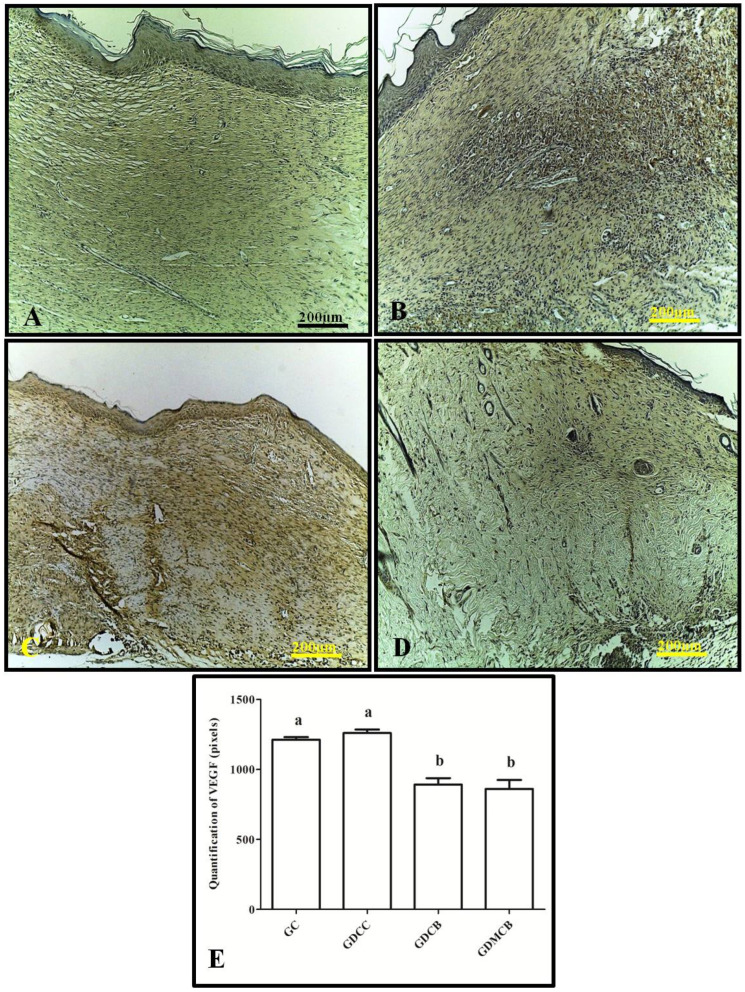
Immunohistochemical analysis for VEGF of wounds from animals at day 14. (**A**) GC group; (**B**) GCC group; (**C**) GDCB group; (**D**) GDMCB group. There is a higher expression in the GC and GDCC groups and a lower expression in the GDCB and GDMCB groups. (**E**) Quantification in pixels. Means followed by the same letter do not differ significantly from each other, as determined via Tukey and Kramer’s multiple comparisons test (*p* > 0.05).

For VEGF, the wounds showed weak staining after 14 days in the GDCB and GDMCB groups, whereas the GC and GDCC groups showed a strong staining pattern, but no statistical difference was found. Regarding the quantification of VEGF expression in pixels, we observed a significant decrease in pixels in the GDCB and GDMCB groups compared to the other groups (Figure 3).

**Figure 4:**
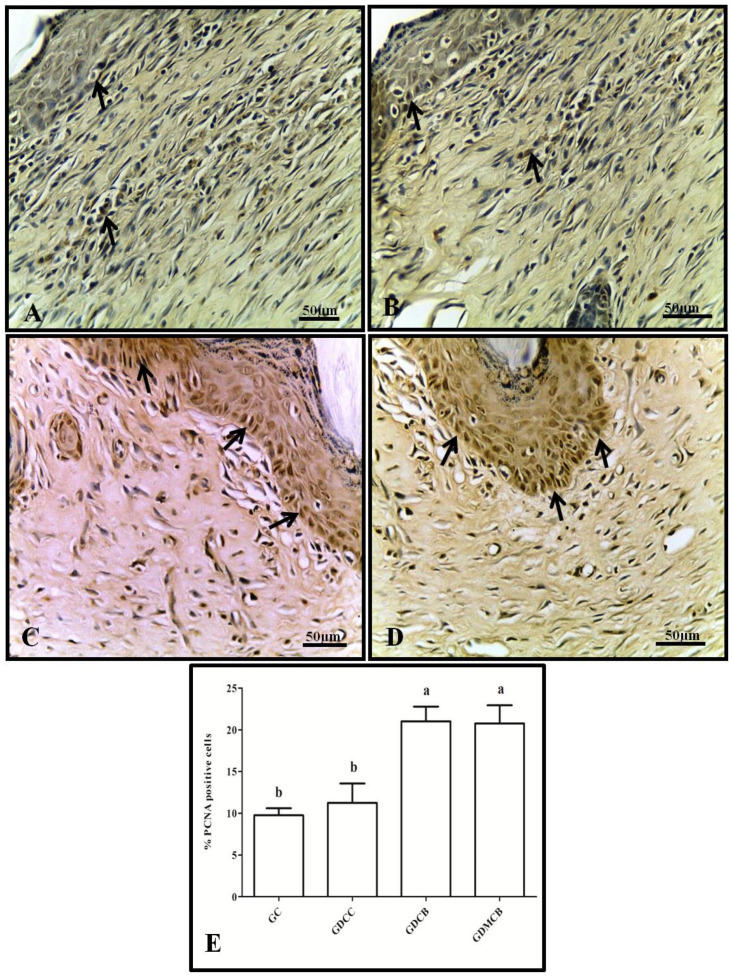
Immunohistochemical analysis for PCNA of wounds from animals at day 14. (**A**) GC; (**B**) GCC; (**C**) GDCB; (**D**) GDMCB. There is a strong expression in the GC and GDCC groups and higher rates of proliferation in the GDCB and GDMCB groups. (**E**) Quantification in pixels. Means followed by the same letter do not differ significantly from each other, as determined via Tukey and Kramer’s multiple comparisons test (*p* > 0.05).

Regarding cell proliferation, PCNA was marked in all groups in the epithelial layer, and marking was weak after 14 days in the GC and GCC groups, without statistical difference, whereas the GDCB and GDMCB groups showed a strong staining pattern but no statistical difference. Regarding the quantification of positive PCNA cells, we observed an increase in the proliferation indices, along with a significant increase in pixels in the GDCB and GDMCB groups compared to the other experimental groups (Figure 4).

### 3.2. Evaluation and Quantification of Collagen I and III

**Figure 5:**
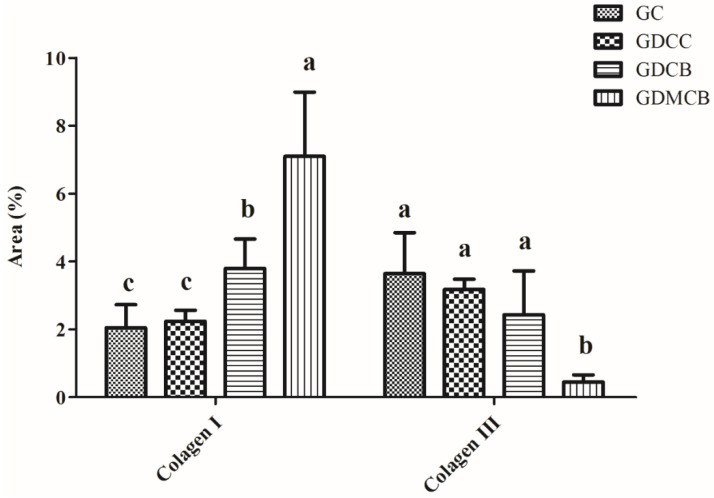
Quantification of collagen I and III of wounds from animals at day 14. There are higher rates of proliferation in the GDCB and GDMCB groups. Means followed by the same letter do not differ significantly from each other, as determined via Tukey and Kramer’s multiple comparisons test (*p* > 0.05).

Quantification of type I collagen (mature collagen) revealed a larger concentration (by area quantification) in the GDCB and GDMCB groups, and quantification of type III collagen (immature) revealed a smaller concentration in the GDMCB group at 14 days after operation (Figure 5).

**Figure 6:**
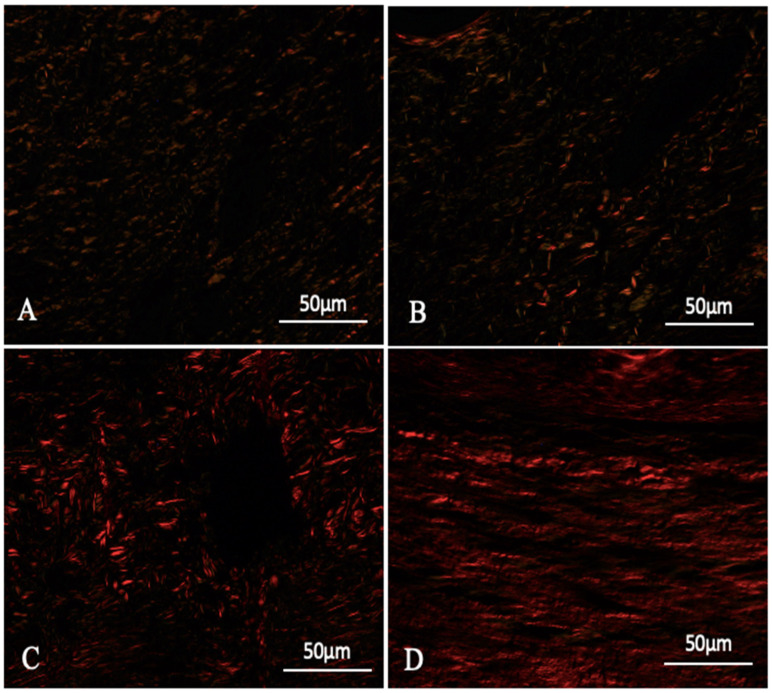
Photomicrographs of the deposition and organization of collagen fibers in skin lesions from animals evaluated 14 days after diabetes induction and respective treatments. (**A**) GC; (**B**) GDCC; (**C**) GDCB; and (**D**) GDMCB. There is a higher concentration of type III collagen fibers (yellow green) in (**A**,**B**) and a higher concentration of type I collagen fibers (red) in (**C**,**D**). For staining, Picrosirius Red was used, and visualization was carried out via polarized light microscopy.

The evaluation of the collagen fibers showed that the GDMCB group presented organized thick type I collagen fibers parallel to the surface, whereas the GC and GDCC groups, after 14 days, still presented a predominance of type III collagen fibers in fragmented and randomly arranged forms and few type I fibers (Figure 6).

## 4. Discussion

Here, we demonstrate that the application of melatonin in conjunction with the use of a bacterial cellulose dressing significantly accelerates skin repair in an experimental model of diabetes. The combined treatment appears to mediate the production of cytokines and collagen, both critical elements in wound healing, especially in diabetic conditions where repair processes are often compromised. By analyzing the effects of this combination therapy, we provide new insights into the mechanisms underlying tissue repair in a hyperglycemic environment.

### 4.1. Bacterial Cellulose in Wound Healing

The bacterial cellulose hydrogel presented in this study was produced microbiologically using the bacterium *Gluconacetobacter hansenii*, with sugarcane molasses (*Saccharum officinarum*) being used as the raw material. From this bacterial cellulose hydrogel, many biomedical byproducts can be generated, such as perforated membranes, intact membranes, tubes, films, and sponges. These byproducts can be used for various purposes, such as urethral reconstruction, tympanic membrane perforation, and other applications [14].

Bacterial cellulose films were used on animals with skin wounds of various sizes, caused by different traumas or tumor excisions requiring healing by secondary intention. In this study, it was observed that granulation tissue demonstrated accelerated growth in the initial phase of re-epithelialization, occurring, on average, by the fifth day after the start of treatment, and the granulation tissue filled the entire wound space. Regarding the healing time, all wounds treated with the sugarcane-derived biopolymer progressed without complications, showing a shorter healing time compared to wounds of similar size [11].

In more recent studies involving humans, bacterial cellulose was used as a dressing in the form of membranes for the treatment of varicose ulcers. The size of the dressing varied from 2 × 2 cm to 6 × 60 cm, and the thickness varied from 0.01 to 0.02 mm. This study compared the use of bacterial cellulose with a control group that received conventional treatment with triglyceride oil. The efficacy of the treatment was evaluated based on the degree of wound healing, the size of the wound area, tissue characteristics during the healing process, and the number of fully healed wounds. This study demonstrated that the bacterial cellulose membrane has ideal properties as a dressing, as it maintains moisture in the wound bed, absorbs excess exudate, limits infectious processes, and protects the wound from mechanical trauma [12].

In a new study, perforated membranes coupled with a sponge, both made of bacterial cellulose biopolymer, were used in the form of sterile films measuring 10 × 8 cm and 0.01 to 0.02 mm in thickness for the treatment of adult patients with pressure ulcers [13]. The primary outcome assessed was the healing process (tissue characteristics), and the secondary outcome was the healing time. It is important to highlight that no signs of toxic reactions associated with the bacterial cellulose biopolymer were observed, demonstrating that it is a non-toxic and biocompatible dressing. The membrane displayed the essential characteristics of an ideal dressing, making it a potential option for wound coverage. The authors concluded that the bacterial cellulose membrane was effective in treating pressure ulcers, as it acted as a granulation tissue inducer, reducing the depth of the wound. Being an innovative, low-cost dressing, it represents an important therapeutic alternative for this challenging condition.

### 4.2. Melatonin in Wound Healing

Melatonin (N-acetyl-5-methoxytryptamine) is the primary product of the pineal gland, although it can also be secreted by other organs, such as the retina and skin. It has been shown to exert anti-inflammatory effects and is beneficial for the treatment of cardiovascular diseases, neurological disorders, and obesity-related diabetes. One study prepared injectable hydrogels loaded with melatonin and investigated their effects in a full-thickness wound model featuring rats [15]. When compared to the control group and a hydrogel without melatonin, the melatonin-loaded hydrogel significantly increased the wound closure percentage, promoted granulation tissue proliferation and re-epithelialization, accelerated collagen deposition, and enhanced angiogenesis and collagen III formation, among other healing factors. These results suggest that the melatonin-loaded hydrogel promoted granulation tissue formation and accelerated wound healing.

An in vitro study demonstrated that melatonin promotes the healing of diabetic wounds by regulating the activity of primary keratinocytes derived from rats [17]. The authors suggested that melatonin reduced high glucose-induced mRNA expression and the release of pro-inflammatory cytokines, including tumor necrosis factor-α, interleukin (IL)-1β, IL-6, and IL-8, in keratinocytes. They also stated that melatonin inhibited oxidative stress and that a high glucose-induced reduction in keratinocyte migration and proliferation, along with increased apoptosis, were counteracted by melatonin treatment. Collectively, these results suggest that melatonin is a potential therapeutic strategy for improving impaired diabetic wound healing by regulating keratinocyte activity.

Finally, a recent study aimed to evaluate the wound healing potential of melatonin when applied to diabetic rat wounds. In this study, a melatonin-containing formulation was developed and topically applied to skin wounds in diabetic rats [19]. The stability of melatonin in the formulation, macroscopic analysis results and the wound contraction index, histological analysis results, and collagen content were evaluated. The study concluded that topical application of melatonin optimized skin wound healing in diabetic rats by attenuating the inflammatory process and promoting an early increase in mononuclear cells at the wound site. The treatment accelerated cutaneous wound contraction, facilitated early collagen fiber maturation, increased collagen deposition, and promoted faster scar connective tissue formation.

### 4.3. The Role of Cytokines in Wound Healing

Cytokines play a pivotal role in the regulation of the wound healing process. IL-6, TNF-α, and VEGF are some of many cytokines released during tissue repair and inflammation. These pro-inflammatory cytokines are essential for orchestrating the complex interplay of immune cells, growth factors, and structural proteins that drive tissue regeneration. IL-6, for instance, exhibits dual functions depending on the stage of healing. During the early phases of repair, IL-6 exerts chemoattractive effects on neutrophils, promoting their migration to the wound site and facilitating the clearance of pathogens and debris. In the later stages, IL-6 promotes keratinocyte proliferation, contributing to the re-establishment of the epidermal layer [25]. Similarly, TNF-α, another critical cytokine, stimulates endothelial cells to produce nitric oxide (NO) and express adhesion molecules, which are essential for leukocyte recruitment and activation at the injury site [25].

Our findings indicated that the expression of IL-6 and TNF-α was significantly reduced in the group treated with both melatonin and bacterial cellulose at the end of the experiment. This decrease can be attributed to the anti-inflammatory and immunomodulatory effects of melatonin, as shown by previous studies [18,20]. Melatonin negatively regulates pro-inflammatory cytokines such as IL-6 and tumor necrosis factor-α, preventing the translocation of nuclear factor kappa B (NF-κB), a transcription factor that drives the production of these pro-inflammatory cytokines. By preventing NF-κB translocation to the nucleus and its binding to DNA, melatonin effectively downregulates the expression of these cytokines, thus attenuating inflammation [18,26]. These findings align with previous studies that have highlighted melatonin’s ability to suppress the inflammatory response, particularly in hyperglycemic conditions.

VEGF, a key angiogenic factor, is also crucial for wound healing, particularly in diabetic patients, where impaired angiogenesis is a major obstacle to effective repair. VEGF can be activated by hyperglycemia. Interestingly, our study results revealed a lower quantification of VEGF in the GDCB and GDMCB groups at 14 days after operation. This reduction in VEGF expression likely reflects the advanced stage of wound healing in these groups, where angiogenesis is no longer required because the wound has nearly healed. This supports the hypothesis that a defect in endogenous VEGF production may contribute to the impaired reparative angiogenesis observed in diabetic wounds. Previous studies have shown that the administration of VEGF can improve healing outcomes in diabetic models, further underscoring its role in restoring proper vascularization in impaired wounds [7,27,28].

### 4.4. Cell Proliferation

Beyond cytokine modulation, our study highlights the influence of bacterial cellulose and melatonin on cell proliferation, particularly in the context of keratinocyte activation and re-epithelialization. Bacterial cellulose hydrogel has been shown to promote cell proliferation, enhance in vitro cell viability, support the differentiation of mesenchymal stem cells, and help in reprogramming the essential macrophage population to promote cell proliferation and tissue repair, facilitating the transition from the inflammatory phase of healing to the proliferative phase of healing [29,30,31,32,33]. Studies have also shown that both bacterial cellulose hydrogel and melatonin accelerate re-epithelialization by activating keratinocytes, a critical step in the wound healing process [17,33,34,35,36,37,38]. In our study, we observed enhanced epithelium labeling, suggesting that both the bacterial cellulose hydrogel and melatonin, whether applied individually or in combination, may act synergistically to improve keratinocyte function, thereby facilitating closure of diabetic wounds. These findings provide promising evidence that this combination therapy could be particularly beneficial in the treatment of chronic wounds in diabetic patients, where re-epithelialization is often delayed.

### 4.5. The Role of Collagen in Wound Healing

Collagen is essential in wound healing because it provides structural integrity to the newly formed tissue. During the healing process, the initial granulation tissue rich in collagen type III and blood vessels is gradually replaced by collagen type I and a few blood vessels, resulting in tissue that more closely resembles the normal dermal structure of healthy individuals, where collagen type I constitutes approximately 80% of the collagen content in the extracellular matrix of the dermis, while type III collagen accounts for about only 10% [39]. The present study showed an increase in the amount and thickness of type I collagen at the end of the experiments in the GDMCB group compared to the other groups. This observation is consistent with previous reports that bacterial cellulose can enhance collagen deposition, supporting the maturation of granulation tissue into a more stable and organized dermal matrix [11,40,41]. Furthermore, melatonin has been shown to enhance collagen synthesis, particularly in diabetic models, by mitigating oxidative stress and promoting fibroblast activity [15,19,42]. The combined effects of bacterial cellulose and melatonin in our study suggest that this therapeutic strategy not only accelerates wound closure but also improves the quality of the regenerated tissue, ensuring stronger and more resilient skin.

## 5. Conclusions

It can be concluded that treating skin lesions of diabetic rats supplemented with melatonin using a bacterial cellulose-based dressing has positive effects in regulating the expression of inflammatory cytokines, vascular endothelial growth factor, and collagen, showing that this association may be a possible therapy approach in wound healing.

By modulating cytokine expression, promoting cell proliferation, and improving collagen deposition, this combination therapy addresses several of the key factors that contribute to impaired healing in diabetes. These findings pave the way for further research into the clinical potential of melatonin and bacterial cellulose in the treatment of chronic wounds, particularly in patients with diabetes.

## Data Availability

The original contributions presented in the study are included in the article, further inquiries can be directed to the corresponding authors.
